# A Randomized Controlled Trial of a Positive Family Holistic Health Intervention for Probationers in Hong Kong: A Mixed-Method Study

**DOI:** 10.3389/fpsyg.2021.739418

**Published:** 2021-12-07

**Authors:** Agnes Y.-K. Lai, Shirley M.-M. Sit, Carol Thomas, George O.-C. Cheung, Alice Wan, Sophia S.-C. Chan, Tai-hing Lam

**Affiliations:** ^1^School of Nursing, The University of Hong Kong, Pokfulam, Hong Kong SAR, China; ^2^School of Public Health, The University of Hong Kong, Hong Kong, Hong Kong SAR, China; ^3^Hong Kong Social Welfare Department, Hong Kong, Hong Kong SAR, China

**Keywords:** probationer, community-based, positive psychology, theory-based, physical activity, Zero-time exercise, family communication

## Abstract

**Introduction:** Probationers, offenders with less serious and non-violent offences, and under statutory supervision, have low levels of self-esteem and physical health, and high level of family conflict, and poorer quality of family relationships. This study examined the effectiveness of the existing probation service and the additional use of a positive family holistic health intervention to enhance physical, psychological, and family well-being in probationers and relationships with probation officers.

**Methods:** Probationers under the care of the Hong Kong Social Welfare Department were randomized into a care-as-usual control group (CAU), a brief intervention group (BI) receiving two 1-h individual sessions [of a brief theory-based positive family holistic health intervention integrating Zero-time Exercise (simple and easy-to-do lifestyle-integrated physical activity) and positive psychology themes of “Praise and Gratitude” in the existing probation service], or a combined intervention group (CI) receiving BI and a 1-day group activity with family members. The outcomes were physical activity, fitness performance, self-esteem, happiness, anxiety and depression symptoms, life satisfaction, quality of life, family communication and well-being, and relationships with probation officers. Self-administered questionnaires and simple fitness tests were used at baseline, 1-month and 3-month follow-up. Linear mixed model analysis was used to compare difference in the changes of outcome variables among groups, adjusted of sex, age, and baseline values. Focus group interviews were conducted. Thematic content analysis was used.

**Results:** 318 probationers (51% male) were randomized into CAU (*n* = 105), BI (*n* = 108), or CI (*n* = 105) group. CAU showed enhanced physical activity, fitness performance and psychological health, and family communication with small effect sizes (Cohen’s d: 0.19–0.41). BI and CI showed further improved physical activity, family communication and family well-being (Cohen’s d: 0.37–0.70). Additionally, CI reported greater improvements in the relationships with probation officers than CAU with a small effect size (Cohen’s d: 0.43). CI also reported greater increases in physical activity and family communication than BI with small to moderate effect sizes (Cohen’s d: 0.38–0.58). Qualitative feedbacks corroborated the quantitative findings.

**Conclusion:** Our trial provided the first evidence of the effectiveness of probation service and the additional use of an innovative, relatively low-cost, theory-based brief positive family holistic health intervention. This intervention may offer a new model for enhancing probation service.

**Trial Registration:** The research protocol was registered at the National Institutes of Health (identifier: NCT02770898).

## Introduction

Probationers often have low self-esteem and physical health (Center for Substance Abuse Treatment, 2005), and experience higher levels of family conflict and strained family relationships (Comfort, 2016). Reviews have shown probationers have a high risk of mental health problems and suicide (Kolb, 2015; Skinner and Farrington, 2020). Given such vulnerabilities, there is a need to strengthen and promote a healthy lifestyle among probationers to enhance individual and family well-being.

Within the Hong Kong criminal justice system, the Hong Kong probation service is a community-based rehabilitation program that emphasizes the enabling of offenders to reform rather than “controlling, punishing or monitoring” (Chui, 2004) and offers statutory supervision for offenders who are put on probation and community service order (Social Welfare Department, 2021). Probationers are first and second offenders whose current offences are less serious and non-violent, and placed under statutory supervision of a probation officer for a specified period of time. The goals of probation service are to prepare probationers to re-integrate into the community and enhance their holistic health, including both personal and family domains. Evaluating the impact of probation service on probationers is necessary for both the effective practices of probation officers and the assessment of the success of their work. There are limited studies that have evaluated the effectiveness of probation service on social and behavioral changes in probationers in Hong Kong (Chui, 2003; Chui and Chan, 2013) and elsewhere (Sexton and Turner, 2010; Jeon et al., 2021). Two studies explored the subjective views on and experiences of probation supervision among young adult offenders (Chui, 2003), and juvenile probationers’ perceptions of probation officers as social workers in Hong Kong (Chui and Chan, 2013), respectively. Two additional studies examined the effectiveness of family functioning therapy (Sexton and Turner, 2010) and a forest therapy program (Jeon et al., 2021) in juvenile probationers.

Family holistic health focuses on the interactive, functional, psychosocial, and health processes of the family experience and encompasses wellness and illness variables (Ho et al., 2019). The increasingly complex and diverse family structure has led to significant concerns for the well-being of families in Hong Kong (Lam et al., 2012). Unhealthy family environments, such as high levels of disruption and conflict, also place family members at greater risk for problematic behaviors. On the contrary, strong and healthy family relationships can have a positive influence on well-being (Galvin et al., 2015), and social support from family members can serve as a protective factor against problematic behaviors (Thomas et al., 2017). Thus, interventions that increase protective factors and reduce risk factors among probationers are needed.

Positive psychology is a science of happiness that focuses on positive emotions and personal strengths (Seligman and Flourish, 2012). A meta-analysis of 51 positive psychology interventions concluded that positive psychology interventions significantly enhanced psychosocial well-being (Sin and Lyubomirsky, 2009). “Praise and Gratitude” is a combination of the expression of thankfulness and an emotional sense of appreciation (Emmons and McCullough, 2003; Peterson and Seligman, 2004), which are among the easiest and most commonly applied positive psychology themes into daily life to enhance personal and family well-being (Ho et al., 2019; Lai et al., 2020).

Physical activity is an essential component of well-being and helps reduce anxiety, stress, and depression, and improve self-esteem and psychological well-being (Sonstroem and Morgan, 1989; Strauss et al., 2001). Our team created “Zero-time exercise” (ZTEx), a new approach to integrate simple strength- and stamina-enhancing physical activity into daily life. ZTEx does not require extra time, money, and equipment and can be done anytime, anywhere and by anybody (Lai A. et al., 2019). ZTEx uses a foot-in-the-door approach to encourage individuals to start exercising in small steps through building exercise self-efficacy. This approach is consistent with American physical activity guidelines that moving more and sitting less is beneficial for nearly everyone, and that some physical activity is better than none (Piercy et al., 2018). ZTEx is an innovative, creative, and fun family activity, where family members of all ages can create and compete in friendly exercise games (Lai et al., 2020). Examples of ZTEx while sitting and standing include pedaling both legs and standing on one leg, respectively, with more examples shown in our YouTube videos.^1^

The citywide Jockey Club FAMILY Project launched in 2008 was initiated and funded by The Hong Kong Jockey Club Charities Trust. The project, conducted in collaboration with the School of Public Health of The University of Hong Kong (HKU-SPH), aimed to promote family well-being in Hong Kong families. We integrated ZTEx and positive psychology into various community-based programs for different populations (e.g., low-income families, parents, children, and elderly), with consistently positive impacts on family communication and personal and family well-being (Lai et al., 2018, 2020; Lai A. et al., 2019; Lai Y. et al., 2019; Ho et al., 2020).

Under the FAMILY Project, HKU-SPH was invited by the Social Welfare Department (SWD) of the Hong Kong SAR Government to collaborate in the design, implementation, and evaluation of the existing probation service. The current trial used an innovative, relatively low-cost, theory-based positive family holistic health intervention based on the Social Learning Theory, with an emphasis on the interaction among individual, behavioral, and environmental factors that allow individuals to learn by observing and imitating the behaviors of others (Akers and Jennings, 2019).

**FIGURE 1:**
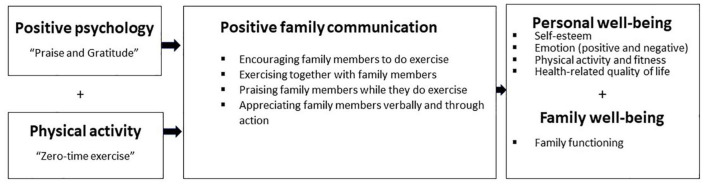
The conceptual framework of the family holistic health intervention.

Our intervention integrated the positive psychology themes of “Praise and Gratitude” of positive psychology with a simple, lifestyle-integrating physical activity (ZTEx) to focus on (i) enhancing probationers’ healthy lifestyle (physical activity), personal well-being (self-esteem, emotions, physical fitness and quality of life), and (ii) encouraging them to interact with family members with positive family communication with the aims of strengthening social bonds and improving family well-being. Figure 1 shows the conceptual framework of the intervention.

Our search of PubMed and Web of Science using a combination of keywords including “exercise,” “physical activity,” “intervention,” “RCT,” “family,” and “happiness” up to 30 June 2021 yielded only one study on an exercise intervention RCT on mother-child dyads to improve sedentary behavior and exercise enjoyment (Tuominen et al., 2020), and our team’s previous RCT study on integrating physical activity to improve positive family communication and perceived health in deprived families in Hong Kong (Lai et al., 2020). To the best of our knowledge, we found no reports of RCTs with a family-based physical activity intervention to enhance personal and family well-being.

We hypothesized that probationers in the brief and combined intervention groups would show significantly greater increases in physical activity and improvements in family communication, and personal and family well-being than the care-as-usual control group. This paper reports the development and preliminary evidence on the effectiveness of the existing probation service in Hong Kong and the additional use of a family holistic health intervention on the well-being of probationers.

## Materials and Methods

### Design

This study was a 3-group randomized controlled trial (RCT) with a 3-month follow-up. Participants were randomized into either the “Care-as-usual control group (CAU),” “Brief intervention group (BI),” or “Combined intervention group (CI)” at a 1:1:1 ratio by creating a random sample in Microsoft Excel. The randomization sequence was generated by a research staff who was not involved in the recruitment process, intervention, or data collection.

### Participants

Participants for the study were recruited from probationers under the Probation and Community Service Orders with supervision and guidance from the main and sub-offices of the SWD Eastern Probation and Community Service Orders Office (SWD-PO) from April 2015 to March 2017. The inclusion criteria were: (i) under probation order at the time of recruitment, (ii) aged 13 years or above, with parental consent from those under 18; (iii) with 6 months of remaining probation term; (iv) with family members who are in Hong Kong; and (v) with basic literacy skills with the ability to comprehend and complete the evaluation questionnaires. The exclusion criteria were: (i) with active severe psychiatric problems, developmental and intellectual disabilities; and (ii) those who committed sexual offences. Recruitment was ongoing during the span of the project as new probation and community service orders were received each month. Participation was entirely voluntary, and participants had the right to withdraw at any time without any consequences. Written consent was required from the participants prior to the study.

### Intervention

#### Working Committee and Training for Probation Officers

A working committee comprising public health academics (a medical officer and a nurse) and 3 registered social workers co-designed the intervention and evaluation questionnaires, and refined them after obtaining feedback from other probation officers. Before designing the program for probationers, a needs assessment was first conducted with probation officers to identify the perceived needs of probationers and the feasibility and challenges for program implementation and evaluation. Then, a 2-day train-the-trainer workshop (TTT) (with four sessions) for the probation officers was conducted. On the first day, the first session was to introduce ZTEx and allow them to experience the integration of ZTEx into daily life. The second session was to explain the rationale of the holistic health intervention and the expected role of the probation officers in the program. The third and fourth sessions were conducted one month after the first two sessions, with the aims of strengthening the competence and attitudes in relation to ZTEx, briefly introducing the integration of positive psychology into the program, and explaining the logistics for probationers. Findings from the TTT showed that ZTEx effectively enhanced physical activity and improved the fitness of probation officers, with details reported in our sister paper (Lai A. et al., 2019). A practice manual was given to each probation officer to reiterate the concepts of positive psychology and serve as a reference guide for the implementation of the community-based intervention. Probation officers were randomly allocated to their responsible groups to conduct the same intervention until the end of the entire program, with each receiving a checklist for implementation. This arrangement was to ensure the fidelity of the intervention.

#### The Community-Based Positive Family Holistic Health Intervention for Probationers

##### Care-as-Usual Control Group

As a control group, participants in CAU received the usual probation service, which was a one-hour monthly meeting with their probation officers. The content of the usual probation service was to discuss general issues in relation to their daily lives and relationships with family members. Participants were offered the combined intervention and souvenir packs (including a handgrip and towel) after completing the 3-month assessments.

##### Brief Intervention Group

Participants in BI also received the usual probation service, but the first two one-hour monthly meetings upon joining the program were the individual brief positive family holistic health, which was run by the trained probation officers. It aimed to promote (i) participants’ knowledge, intention, and behaviors related to physical activity, particularly ZTEx, (ii) changes in behavior by setting goals and formulating realistic outcome expectancies, and (iii) family relations and well-being by praising and exercising with family members. Table 1 shows the content outline of the brief individual intervention.

**TABLE 1:** The content outline of individual brief intervention and group activity of combined intervention.

A. Content outline of the individual brief intervention		
Session one (at baseline)		
Duration	Steps	Goals
20 min	• Introduce the age- and sex-specific fitness reference values and discuss the clinical relevance. • Encourage the participants to compare the normative data with their own results.	To assess their own health and enhance the knowledge of the harmful effects of sedentary behavior and benefits of physical activity. To enhance intention of reducing sedentary behavior and increasing physical activity.
20 min	• Introduce Zero-time exercise (ZTEx), demonstrate the examples of different movements and do the exercise together with participants. • Share personal experiences and benefits of doing physical activity, particularly ZTEx.	To enhance their knowledge and self-efficacy in relation to ZTEx. To strength the motivation and promote its conversion to action.
20 min	• Invite participants to set realistic goals and plan for actions and introduce the workbook to participants. • Introduce the importance of “Gratitude and appreciation,” encourage to share what has been learnt and communicate with family.	To help set action plan and goals. To enhance positive family communication and well-being.
**Session 2 (at 1 month after session 1)**		
20 min	• Invite participants to share their experience in relation to physical activity, ZTEx and family communication. • Review the records of their workbook.	To monitor the progress. To review and enhance their motivation.
20 min	• Discuss the barriers encountered in doing physical activity and explore the solution with participants. • Highlight their successfulness in exercising and positive family communication.	To enhance self-efficacy. To provide positive reinforcement.
20 min	• Provide encouragement and support. • Conclude with a summary and key statements.	To strengthen exercise motivation and regulatory factors.
**B. Content outline of the group activity of the combined intervention (4 ho and 30 min)**		
30 min	• Answer the questionnaire and perform fitness assessments at baseline.	To provide an ice-breaking activity and increase participants’ health awareness and interest that followed.
45 min	• Receive an Interactive seminar on physical activity, particularly in ZTEx.	To introduce ZTEX by health professionals and proactively invite participation in the intervention.
45 min	• Conduct family Interactive physical activity games.	To provide good family interaction time and invite exercising with family members.
60 min	• Lunch.	
120 min	• Conduct positive psychology-based family session.	To encourage participants to express appreciation to family members.
20 min	• Participants sharing session.	To allow participants to reflect their feeling and the learnt during the group activity.
10 min	• Closing remarks.	

##### Combined Intervention Group

Participants in CI also received the same individual brief intervention with an addition of a one-day 4.5-h group activity. Table 1 shows the content outline of the 2-session group activity. The first session in the morning was an interactive seminar on ZTEx conducted by a medical professional (THL, the founder of ZTEx) and theme-based interactive family games conducted by social workers. The second session in the afternoon was a positive psychology-based family session conducted by social workers. Participants were invited to join the group activity with one of their family members before starting the first individual brief intervention session. The group activity led by probation officers created a supportive environment for positive family time and communication and encouraged the engagement in physical activity with family members through role modeling and peer support.

Each participant in BI and CI was given a workbook to set their goals of engaging in physical activity by themselves and with family members, record their daily physical activity and track their exercise progress over 3 months. The workbook stated the benefits of regular physical activity and the harmful effects of physical inactivity (e.g., the relationship between sedentary behavior and cancer). It was an essential tool to share the learned information with family members and provide valuable tips (e.g., positive communication, praise, and appreciation) to enhance family relationships.

### Data Collection

Self-administered questionnaires and physical fitness were assessed at baseline, 1-month and 3-month follow-up. Physical fitness assessments included single-leg stance and 30-s chair stand tests at all three-time points. Three 1-h focus group interviews were conducted with 24 probationers to obtain their feedback after completing the 3-month follow-up assessment on 12 March 2017 on the main campus of The University of Hong Kong. Probationers’ feedback on the quality of intervention content was collected to triangulate the qualitative and quantitative findings.

### Measures

#### Physical Activity and Fitness

Participants’ engagement in simple strength and stamina-enhancing physical activity while seated and standing was assessed by asking two questions on the number of days the participant engaged in physical activity during the last 7 days; responses ranged from “0” to “7” days, which had been used in our previous study (Lai et al., 2018). Questions from the short form of the International Physical Activity Questionnaire—Chinese version (IPAQ-C) were used to assess participants’ physical activity by asking for the number of days they engaged in at least 10 min of moderate and vigorous physical activity, respectively. The questions were: “During the last 7 days, on how many days did you do at least 10 min of moderate physical activity?”; and “During the last 7 days, on how many days did you do at least 10 min of vigorous physical activity?” The internal reliability of the Chinese version of the questionnaire was high, with an intraclass correlation coefficient of 0.79 (Macfarlane et al., 2007).

The lower limb muscular endurance was assessed using a 30-s chair stand test by recording the number of stands completed from the chair in 30-s (Jones et al., 1999). Balance was assessed using a single-leg-stance test by recording the stance duration in which balance on one leg is effectively achieved (for a maximum of 120-s) (Newton, 1989). Questions about general health were asked before the physical fitness assessments. All participants completed these assessments with no reports of discomfort or complaints.

#### Psychological Well-Being and Quality of Life

##### Rosenberg Self-Esteem Scale

The 10-item Rosenberg Self-esteem Scale was used to measure self-esteem. Each question was a score from 1 to 4, with higher scores indicating higher self-esteem (Rosenberg, 1965). The Cronbach’s alpha ranged from 0.80 to 0.84 across three-time points, indicating good reliability.

##### Subjective Happiness Scale

The 4-item Subjective Happiness Scale was adopted to measure subjective happiness. Responses were given on a 7-point Likert scale from 1 (less happy) to 7 (more happy), with a higher total score indicating a higher level of happiness (Lyubomirsky and Lepper, 1999). The Chinese version of the scale has been previously translated and validated in Hong Kong (Nan et al., 2014). The scale demonstrated good internal consistency (Cronbach’s alpha ranged from 0.79 to 0.94), indicating good reliability.

##### Patient Health Questionnaire

The 4-item Patient Health Questionnaire (PHQ-4) was used to assess depression and anxiety. Responses were given on a scale of 0 to 3, with lower scores indicating a lower likelihood of being depressed or anxious (Kroenke et al., 2009). The Cronbach’s alpha ranged from 0.88 to 0.91 across three time points, indicating good reliability.

##### Satisfaction With Life Scale

The 5-item Satisfaction with Life Scale was used with responses given on a 7-point Likert scale from 1 (strongly disagree) to 7 (strongly agree), with a higher total score indicating a higher level of satisfaction with life (Diener et al., 1985). The Cronbach’s alpha ranged from 0.93 to 0.95 across three-time points, indicating good reliability.

##### Short Form Health Survey

The 12-item Short-Form Health Survey (SF-12v2) was used to assess the quality of life, consisting of both mental and physical quality of life. Responses were made on a 3-point scale (1 = “yes, limited a lot” to 3 = “no, not limited at all”) or a 5-point scale (1 = “not at all” to 5 = “extremely”) (Ware et al., 1996). The Chinese version of the scale has been validated in local populations with satisfactory content and criterion validity (Lam et al., 2005).

#### Family Communication

Four outcome-based questions were used to measure the frequency of behavior indicators of family communication, including doing physical activity with family members, praising family members to do physical activity, and expressing appreciation to family members verbally and through action in the last 4 weeks. Responses were made on a scale of 1 (never) to 5 (always), with higher scores indicating more of the target behavior. Self-reported single-item measures of physical activity have been widely used in healthy adult populations (Silsbury et al., 2015).

#### Family Well-Being

The 5-item Family APGAR scale was used to measure the five areas of family function (well-being), including adaptability, partnership, growth, affection, and resolve. A total score of 7–10 suggests a highly functional family, 4–6 suggests a moderately dysfunctional family, and 0–3 suggests a severely dysfunctional family (Smilkstein, 1978). The Cronbach’s alpha ranged from 0.85 to 0.88 across three time points, indicating good reliability.

#### Relationship Between Probationers and Probation Officers

An outcome-based question was used to ask the probationers’ perceived relationship with their probation officers on a 5-point scale, ranging from 1 (poor) to 5 (very good). Higher scores indicated a better relationship.

### Statistical Analysis

Analyses were done using IBM SPSS Statistics 25, with a two-tailed significance of *p* < 0.05. Adhering to intention-to-treat (ITT) principles, all missing values of the outcome variables were substituted by the baseline values. Chi-square analysis was conducted to test if demographic characteristics varied among the CAU, BI, and CI. A mixed-effects model was adopted to investigate the impacts of between-group differences. The intervention group was treated as a fixed effect, and sex, age, and the baseline values of the outcome variables were included as covariates. Estimated marginal means were employed for planned comparisons to examine whether there were within-group differences across time points. The focus group interviews were conducted by an experienced researcher from the working committee. All qualitative interviews were audiotaped and transcribed verbatim in Chinese. Two project members, one of whom had attended the interviews, coded the transcripts, which were analyzed using thematic framework analysis following the guidelines recommended by Morse and Field (1995). A mixed-methods design was used to interrelate and interpret the qualitative and quantitative data (Creswell and Clark, 2017).

## Results

### Participants

Of the 463 eligible probationers invited to join the study, 318 joined and completed the questionnaire and fitness assessment at baseline before the first session. Around half of them were female (48.7%), aged 20–39 years (47.2%), and with about one third married (34%).

**FIGURE 2:**
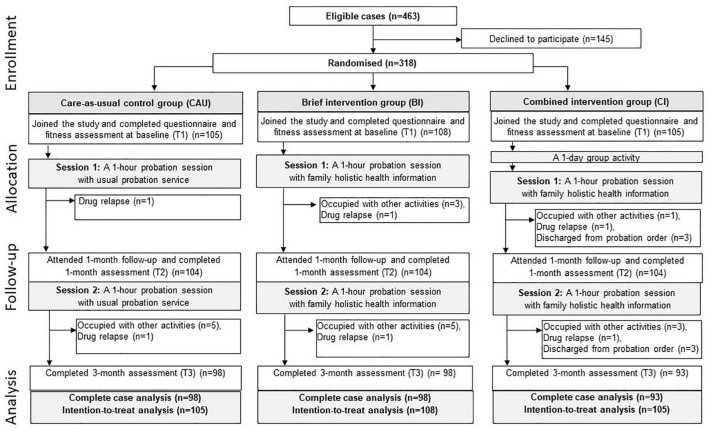
The study flow chart.

About two-thirds had secondary level education (66.7%) and were employed (64.5%). The probationers were allocated into CAU (*n* = 105), BI (*n* = 108), and CI (*n* = 105). Ten probationers (1 from CAU, 4 from BI, and 4 from CI) were absent from the 1-month follow-up, and 19 (6 from CAU, 6 from BI, and 9 from CI) were absent from the 3-month follow-up. The remaining 279 probationers completed the assessments at all time points. Figure 2 shows the recruitment and study flowchart. Table 2 shows no significant differences in the baseline characteristics among the three groups.

**TABLE 2:** Baseline demographic characteristics of probationers (*n* = 318).

	All	CAU	BI	CI	
	***n* = 318**	***n* = 105**	***n* = 108**	***n* = 105**	***p*-value**

		***n* (%)**	***n* (%)**	***n* (%)**	

**Sex**					
Male	163 (51.3)	58 (55.2)	52 (48.1)	53 (50.5)	0.56
Female	155 (48.7)	47 (44.8)	56 (51.9)	52 (49.5)	
**Age**					
12–19	50 (15.7)	24 (22.9)	13 (12.0)	13 (12.4)	0.30
20–39	150 (47.2)	46 (43.8)	52 (48.1)	52 (49.5)	
40–59	85 (26.7)	23 (21.9)	33 (30.6)	29 (27.6)	
≥60	33 (10.4)	12 (11.4)	10 (9.3)	11 (10.5)	
**Marital status** ^a^					
Not married	171 (53.8)	63 (60.0)	50 (46.3)	58 (55.2)	0.30
Married	108 (34.0)	34 (32.4)	43 (39.8)	31 (29.5)	
Separated, divorced, widowed	39 (12.2)	9 (8.6)	14 (13.0)	16 (15.2)	
**Education** ^b^					
Primary or below	42 (13.2)	13 (12.4)	14 (13.0)	15 (14.3)	0.81
Secondary	12 (66.7)	75 (71.4)	71 (65.7)	66 (62.9)	
Post-secondary or above	64 (20.1)	18 (17.1)	22 (20.4)	24 (22.9)	
**Employment** ^c^					
Student	27 (8.5)	15 (14.3)	7 (6.2)	5 (4.8)	0.2
Employed full-time/part-time	205 (64.5)	68 (64.8)	68 (63.0)	69 (65.7)	
Unemployed/retired	42 (13.2)	14 (13.3)	14 (13.0)	14 (13.3)	
Homemaker	44 (13.8)	10 (9.5)	16 (14.8)	18 (17.1)	
**Duration of probation**					
Half year or below	207 (65.1)	68 (64.8)	76 (70.3)	63 (60.0)	0.21
Half year to 1 year	83 (27.4)	27 (25.7)	27 (25.0)	33 (31.4)	
1 to 1.5 years	20 (6.3)	10 (9.5)	3 (2.8)	7 (6.6)	
>1.5 years	4 (1.5)	2 (0.2)	1 (1.0)	1 (1.0)	

*CAU = Care-as-usual control group, BI = Brief intervention group, CI = Combined intervention group.*

*^a^7 missing value, n = 311; ^b^4 missing value, n = 314; ^c^18 missing value, n = 300. No significant difference among three groups.*

Twenty-four probationers joined the focus group interviews after completing the intervention. Half of them were female (50%), nearly half were aged 20–39 years (46%) and about one third were married (34%). About two-thirds had secondary level education or above (66.7%) and were employed (60.8%). 58.3% had less than half a year of probation term remaining. No significant differences in probationers’ characteristics were observed between those who participated in the focus group interviews and those who did not. No harm or unintended effects were detected in either group.

### Changes in Physical Activity

Table 3 shows CAU reported significant increases in days spent engaging in simple strength and stamina-enhancing physical activity (ZTEx) while seated at 1- and 3-month follow-up. BI reported significant increases in days spent engaging in ZTEx while seated and standing at 3-month follow-up. CI reported significant increases in days spent engaging in ZTEx while seated and standing, and moderate physical activity at 1- and 3-month follow-up, and an increase in vigorous physical activity at 1-month follow-up. Effect sizes ranged from small to moderate (Cohen’s d: 0.19–0.50, all *p* < 0.05).

**TABLE 3:** The within-group difference in physical activity, fitness performances, psychological well-being, and quality of life at 1- and 3-month follow-up in three groups: Intention-to-treat analysis.

	CAU (*n* = 105)		BI (*n* = 108)		CI (*n* = 105)	
	Mean ± SD	Cohen’s d	Mean ± SD	Cohen’s d	Mean ± SD	Cohen’s d
**Physical activity**						
**Days spent engaging in physical activity while seated**						
T1	1.8 ± 2.4^##^		2.4 ± 2.6^#^		2.1 ± 2.6^##^	0
T2, (T2 vs. T1)	2.5 ± 2.6	0.26**	2.8 ± 2.4	0.16	2.8 ± 2.4	0.29**
T3, (T3 vs. T1)	2.3 ± 2.4	0.19*	3.2 ± 2.5	0.30**	2.7 ± 2.5	0.26*
**Days spent engaging in physical activity while standing**						
T1	2.1 ± 2.4		2.5 ± 2.6^#^		2.2 ± 2.6^###^	
T2, (T2 vs. T1)	2.2 ± 2.5	0.04	3.0 ± 2.5	0.18	3.1 ± 2.5	0.36***
T3, (T3 vs. T1)	2.4 ± 2.4	0.10	3.3 ± 2.5	0.30*	3.0 ± 2.5	0.33***
**Days spent engaging in moderate physical activity**						
T1	2.2 ± 2.5		2.4 ± 2.5		1.9 ± 2.1^###^	
T2, (T2 vs. T1)	2.0 ± 2.3	–0.09	2.3 ± 2.4	–0.07	3.0 ± 2.4	0.50***
T3, (T3 vs. T1)	2.2 ± 2.5	0.02	2.1 ± 2.3	–0.12	2.7 ± 2.3	0.36***
**Days spent engaging in vigorous physical activity**						
T1	1.3 ± 1.9		1.4 ± 1.8		1.1 ± 1.6^#^	
T2, (T2 vs. T1)	1.2 ± 1.7	–0.05	1.6 ± 2.0	0.08	1.6 ± 1.9	0.30**
T3, (T3 vs. T1)	1.1 ± 1.5	–0.11	1.5 ± 1.9	0.07	1.5 ± 1.8	0.21
**Fitness performance**						
**Single-leg stand test, seconds**						
T1	85.1 ± 40.0		91.8 ± 39.8		84.6 ± 40.1	
T2, (T2 vs. T1)	84.4 ± 38.5	–0.02	90.3 ± 37.7	–0.04	83.9 ± 39.3	–0.02
T3, (T3 vs. T1)	83.6 ± 38.8	–0.05	90.5 ± 37.9	–0.06	81.7 ± 40.2	–0.03
**30-s chair stand test, number of stands**						
T1	19.4 ± 7.9^###^		18.9 ± 7.6^###^		22.4 ± 8.9^#^	
T2, (T2 vs. T1)	21.3 ± 9.0	0.22***	21.3 ± 8.1	0.30***	23.7 ± 9.8	0.14*
T3, (T3 vs. T1)	22.1 ± 9.1	0.32***	21.8 ± 8.4	0.35***	23.5 ± 8.7	0.12*
**Psychological well-being**						
**Self-esteem**						
T1	27.0 ± 4.7		27.0 ± 3.6^#^		27.7 ± 4.1	
T2, (T2 vs. T1)	27.4 ± 4.6	0.08	27.6 ± 3.5	0.16**	27.6 ± 4.6	–0.03
T3, (T3 vs. T1)	27.1 ± 5.1	0.02	27.5 ± 3.7	0.13	27.8 ± 4.2	0.00
**Subjective happiness**						
T1	17.0 ± 4.7		17.7 ± 4.1^###^		17.7 ± 4.8	
T2, (T2 vs. T1)	17.5 ± 4.6	0.11	17.9 ± 4.0	0.05	18.0 ± 4.3	0.07
T3, (T3 vs. T1)	17.7 ± 4.2	0.14	18.9 ± 4.0	0.29***	18.1 ± 4.3	0.09
**Anxiety and depression symptoms**						
T1	2.5 ± 2.6		2.9 ± 2.8		3.0 ± 3.0	
T2, (T2 vs. T1)	2.7 ± 3.0	0.08	2.8 ± 3.0	–0.05	2.4 ± 2.4	–0.21*
T3, (T3 vs. T1)	2.5 ± 2.6	0.03	2.7 ± 2.9	–0.09	2.4 ± 2.5	–0.19
**Life satisfaction**						
T1	20.8 ± 7.4		21.9 ± 7.1		21.6 ± 6.8	
T2, (T2 vs. T1)	21.6 ± 7.5	0.11	21.9 ± 6.5	0.00	22.6 ± 6.8	0.16
T3, (T3 vs. T1)	21.7 ± 7.4	0.12	22.8 ± 6.2	0.14	22.8 ± 6.8	0.18
**Quality of life**						
**Physical quality of life**						
T1	47.4 ± 8.6		47.5 ± 8.5		46.3 ± 9.0	
T2, (T2 vs. T1)	46.8 ± 8.2	–0.07	47.9 ± 8.5	0.04	47.3 ± 8.3	0.12
T3, (T3 vs. T1)	48.2 ± 7.8	0.09	47.3 ± 8.2	–0.02	46.6 ± 8.3	0.04
**Mental quality of life**						
T1	44.3 ± 9.9^#^		44.1 ± 8.5		44.8 ± 8.9	
T2, (T2 vs. T1)	46.4 ± 10.2	0.21**	44.9 ± 9.4	0.09	46.5 ± 9.1	0.19*
T3, (T3 vs. T1)	45.3 ± 10.4	0.10	45.1 ± 8.9	0.11	45.5 ± 8.7	0.08

*CAU = Care-as-usual control group, BI = Brief intervention group, CI = Combined intervention group.*

*T1 = baseline, T2 = 1-month follow-up, T3 = 3-month follow-up.*

*Repeated Measures Analysis of Variance and paired t-test to compare parametric data among three timepoints and between two timepoints, respectively.*

*T2 vs. T1 = values at 1-month follow-up versus values at baseline; T3 vs. T1 = values at 3-month follow-up versus values at baseline.*

Difference among three timepoints: ^#^p < 0.05, ^##^p < 0.01, ^###^p < 0.001; Difference between two timepoints: *p < 0.05, **p < 0.01, ***p < 0.001.

*Effect size (Cohen’s d): small = 0.20, moderate = 0.50, and large = 0.80.*

**FIGURE 3:**
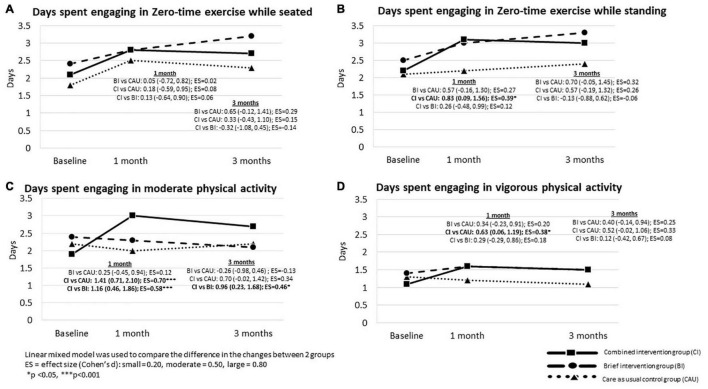
The between –group difference in the changes in physical activity at 1- and 3-month follow-up in three groups: Intention-to-treat analysis.

Figure 3 shows no significant difference in changes in physical activity between BI and CAU. Compared with CAU, CI reported significantly greater increases in days spent engaging in ZTEx while standing by 0.83 days (95% CI: 0.09, 1.56), moderate physical activity by 1.41 days (95% CI: 0.71, 2.10), and vigorous physical activity by 0.63 days (95% CI: 0.06, 1.19) than the CAU at 1-month follow-up with small effect sizes (Cohen’s d: 0.38–0.70, all *p* < 0.05), but not at 3-month follow-up. Compared with BI, CI reported significantly greater increases in days spent engaging in moderate physical activity by 1.16 days (95% CI: 0.46, 1.86) at 1-month follow-up and by 0.96 days (95% CI: 0.23, 1.68) at 3-month follow-up. The effect sizes ranged from small to moderate (Cohen’s d: 0.46–0.58, all *p* < 0.05). No significant difference in the changes in days engaging in ZTEx while seated and standing and vigorous physical activity were reported between BI and CI at 1- and 3-month follow-up.

### Changes in Fitness Performance

Table 3 shows no significant improvement in the duration of the single-leg stand in all three groups. Significant improvements in the number of stands in the 30-s chair stand test were reported for all groups at both 1-month follow-up and 3-month follow-up with small effect size (Cohen’s d: 0.12–0.35; all *p* < 0.05). Table 4 shows no significant differences in the changes in the duration of the single-leg stand and number of stands among three groups at 1- and 3-month follow-up.

**TABLE 4:** The between-group difference in the changes in physical fitness, psychological well-being, and quality of life at 1- and 3-month follow-up in three groups: Intention-to-treat analysis.

	BI vs. CAU		CI vs. CAU		CI vs. BI	

	Mean difference (95%CI)	Cohen’s d	Mean difference (95%CI)	Cohen’s d	Mean difference (95%CI)	Cohen’s d
**Difference in the changes at 1 month**						
**Physical fitness**						
Single-leg stand test, second	1.15 (–7.80, 10.09)	0.04	0.26 (–8.86, 9.38)	0.01	–0.89 (–10.02, 8.24)	–0.03
30-s chair stand test, number of stand	0.28 (–1.46, 2.02)	0.05	–0.41 (–2.23, 1.40)	–0.08	–0.69 (–2.50, 1.11)	–0.13
**Psychological well-being**						
Self-esteem	0.18 (–0.80, 1.16)	0.06	–0.34 (–1.34, 0.65)	–0.12	–0.52 (–1.52, 0.47)	–0.18
Subjective happiness	0.00 (–1.03, 1.03)	0.00	0.10 (–0.95, 1.15)	0.03	0.09 (–0.94, 1.13)	0.03
Anxiety and depression symptoms	–0.18 (–1.00, 0.65)	–0.07	–0.66 (–1.50, 0.17)	–0.27	–0.49 (–1.32, 0.35)	–0.20
Life satisfaction	–0.13 (–1.74, 1.49)	–0.03	0.58 (–1.05, 2.21)	0.12	0.71 (–0.91, 2.32)	0.15
**Quality of life**						
Physical quality of life	0.89 (–1.10, 2.88)	0.15	1.41 (–0.59, 3.41)	0.24	0.52 (–1.50, 2.53)	0.09
Mental quality of life	–1.52 (–4.00, 0.97)	–0.21	–0.40 (–2.91, 2.11)	–0.06	1.12 (–1.40, 3.63)	0.15
**Difference in the changes at 3 months**						
**Physical fitness**						
Single-leg stand test, second	0.68 (–8.80, 10.16)	0.02	–0.52 (–10.22, 9.18)	–0.02	–1.21 (–10.91, 8.50)	–0.04
30-s chair stand test, number of stand	–0.12 (–1.87, 1.63)	–0.02	–1.19 (–3.01, 0.63)	–0.23	–1.07 (–2.89, 0.75)	–0.21
**Psychological well-being**						
Self-esteem	0.47 (–0.50, 1.45)	0.17	0.20 (–0.79, 1.19)	0.07	–0.28 (–1.26, 0.71)	–0.10
Subjective happiness	0.79 (–0.26, 1.83)	0.26	0.19 (–0.87, 1.25)	0.06	–0.60 (–1.64, 0.45)	–0.20
Anxiety and depression symptoms	–0.15 (–0.92, 0.61)	–0.07	–0.38 (–1.16, 0.40)	–0.17	–0.22 (–1.00, 0.55)	–0.10
Life satisfaction	0.39 (–1.30, 2.08)	0.08	0.84 (–0.87, 2.54)	0.17	0.45 (–1.24, 2.14)	0.09
**Quality of life**						
Physical quality of life	–0.35 (–2.48, 1.77)	–0.06	–0.22 (–2.36, 1.93)	–0.03	0.14 (–2.02, 2.30)	0.02
Mental quality of life	–0.07 (–2.62, 2.49)	–0.01	–0.27 (–2.85, 2.30)	–0.04	–0.21 (–2.79, 2.38)	–0.03

*CAU = Care-as-usual control group, BI = Brief intervention group, CI = Combined intervention group.*

*Linear mixed model was adopted to examine the between-group differences.*

### Changes in Psychological Well-Being and Quality of Life

Table 3 shows that CAU reported significant improvements in mental quality of life at 1-month follow-up. BI reported significant improvements in self-esteem at 1-month follow-up, and subjective happiness at 3-month follow-up. CI reported significant reductions in anxiety and depression symptoms and mental quality of life at 1-month follow-up. All effect sizes were small (Cohen’s d: 0.16–0.29, all *p* < 0.05). All three groups had no significant improvements in life satisfaction and physical quality of life at 1- and 3-month follow-up (Table 4).

Table 4 shows no significant differences in the improvements in personal well-being (including self-esteem, subjective happiness, anxiety and depression symptoms, life satisfaction, and mental and physical quality of life) among three groups both at 1- and 3-month follow-up.

At the focus-group interviews after the completion of the program, participants reported feeling more motivated, happier, and healthier than before joining the program.

*“You become more alert after exercising, and once you notice improvements in your physical health, then you will put in more effort into what you think and do.” (Housewife, female, 65 years or above)*

*“I became happier. When I am not happy, I will think about happy things.” (Housewife, female, 55–59 years)*

*“I have become healthier for sure*… *it’s better than not moving.” (Housewife, female, 45–49 years)*

### Change in Family Communication

#### Physical Activity With Family Members

Table 5 shows that all three groups reported significant increases in doing physical activity with family members and praising family members to do physical activity at 1- and 3-month follow-up, with small to large effect sizes (Cohen’s d: 0.21–0.84; all *p* < 0.05).

**TABLE 5:** The within-group difference in family communication, family well-being, and relationship with probation officers at 1- and 3-month follow-up in three groups: Intention-to-treat analysis.

	CAU		BI			CI	
	Mean ± SD	Cohen’s d	Mean ± SD		Cohen’s d	Mean ± SD	Cohen’s d
**Family communication**							
**Did physical activity with family members, score**							
T1	1.7 ± 0.9^###^			1.8 ± 1.0^###^		1.8 ± 0.9^###^	
T2, (T2 vs. T1)	1.9 ± 1.0	0.21*		2.6 ± 1.0	0.81***	2.5 ± 1.0	0.76***
T3, (T3 vs. T1)	2.1 ± 1.0	0.41***		2.7 ± 1.1	0.84***	2.5 ± 1.0	0.71***
**Praised family members to do physical activity, score**							
T1	2.4 ± 1.0			2.3 ± 1.0^###^		2.6 ± 1.0^##^	
T2, (T2 vs. T1)	2.5 ± 1.0	0.11		2.8 ± 1.0	0.46***	2.9 ± 0.9	0.35**
T3, (T3 vs. T1)	2.5 ± 1.0	0.19*		2.9 ± 0.9	0.55***	3.0 ± 1.0	0.38**
**Expressed verbal appreciation to family members, score**							
T1	2.9 ± 1.1			2.9 ± 1.1^#^		2.9 ± 1.0^###^	
T2, (T2 vs. T1)	2.8 ± 1.1	–0.11		3.0 ± 1.0	0.04	3.3 ± 0.9	0.34**
T3, (T3 vs. T1)	2.9 ± 1.0	0.02		3.2 ± 1.0	0.21*	3.4 ± 1.0	0.42***
**Expressed appreciation through action to family members, score**							
T1	2.9 ± 1.0			3.1 ± 1.1		3.0 ± 1.1^##^	
T2, (T2 vs. T1)	2.9 ± 1.0	0.02		3.1 ± 0.9	–0.06	3.3 ± 1.0	0.32**
T3, (T3 vs. T1)	2.8 ± 1.1	–0.06		3.3 ± 1.0	0.14	3.4 ± 1.1	0.37**
**Family well-being, score**							
T1	6.3 ± 2.4			6.5 ± 2.5		6.3 ± 2.5^##^	
T2, (T2 vs. T1)	6.0 ± 2.5	–0.10		6.6 ± 2.5	0.05	7.0 ± 2.3	0.30**
T3, (T3 vs. T1)	6.0 ± 2.7	–0.11		6.9 ± 2.4	0.15	6.8 ± 2.4	0.20*
**Relationship with probation officers, score**							
T1	4.3 ± 0.7			4.3 ± 0.7^#^		4.3 ± 0.7^##^	
T2, (T2 vs. T1)	4.3 ± 0.7	–0.04		4.4 ± 0.6	0.09	4.4 ± 0.6	0.15
T3, (T3 vs. T1)	4.3 ± 0.7	–0.09		4.5 ± 0.6	0.21*	4.5 ± 0.6	0.31**

*T1 = baseline, T2 = 1-month follow-up, T3 = 3-month follow-up.*

*T2 vs. T1 = values at 1-month follow-up versus values at baseline; T3 vs. T1 = values at 3-month follow-up versus values at baseline.*

*Repeated Measures Analysis of Variance and paired t-test to compare parametric data among three timepoints and between two timepoints, respectively.*

Difference among three timepoints: ^#^p < 0.05, ^##^p < 0.01, ^###^p < 0.001; Difference between two timepoints: *p < 0.05, **p < 0.01, ***p < 0.001.

*Effect size (Cohen’s d): small = 0.02, moderate = 0.50, large = 0.80.*

**FIGURE 4:**
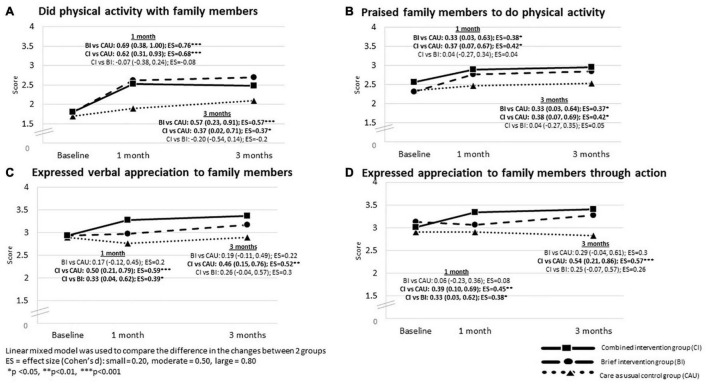
The between–group difference in the changes in family communication at 1- and 3-month follow-up in three groups: Intention-to-treat analysis.

Figure 4 shows, compared with CAU, BI reported significantly greater increases in doing physical activity with family members by 0.69 scores (95% CI: 0.38, 1.00) and 0.57 scores (95% CI: 0.23, 0.91); and praising family members to do physical activity by 0.33 scores (95% CI: 0.03, 0.63) and 0.33 scores (95% CI: 0.03, 0.64), with small to moderate effect sizes (Cohen’s d: 0.37–0.76, *p* < 0.05) at 1- and 3-month follow-up, respectively. The CI also reported significantly greater increases in doing physical activity with family members by 0.62 scores (95% CI: 0.31, 0.93) and 0.37 scores (95% CI: 0.02, 0.71); and praising family members to do physical activity by 0.37 scores (95% CI: 0.07, 0.67) and 0.38 scores (95% CI: 0.07, 0.69) than CAU at 1- and 3-month follow-up, respectively. All effect sizes ranged from small to moderate (Cohen’s d: 0.37–0.68, all *p* < 0.05).

There were no significant differences in the increases in doing physical activity with family members and praising family members to do physical activity between BI and CI at 1- and 3-month follow-up.

#### Expressing Appreciation to Family Members

Table 4 shows no significant increases in expressing appreciation to family members verbally and through action in the CAU at 1- and 3-month follow-up. However, BI reported significant increases in expressing verbal appreciation with a small effect size at 3-month follow-up (Cohen’s d: 0.21, *p* < 0.05), but not at 1-month follow-up. No significant increases in expressing appreciation to family members through action were reported at 1- and 3-month follow-up. CI reported significant increases in expressing appreciation verbally and through action with small effect sizes at 1- and 3-month follow-up (Cohen’s d: 0.32–0.42, all *p* < 0.01).

Figure 4 shows no significant difference in changes in expressing appreciation to family members verbally and through action between BI and CAU at 1- and 3-month follow-up. Compared with CAU, CI reported significantly greater increases in expressing verbal appreciation by 0.50 scores (95% CI: 0.21, 0.79) and 0.46 scores (95% CI: 0.15, 0.76); and expressing appreciation through action by 0.39 scores (95% CI: 0.10, 0.69) and 0.54 scores (95% CI: 0.21, 0.86) with small to moderate effect sizes (Cohen’s d: 0.45–0.59) at 1- and 3-month follow-up, respectively. Compared with the BI, the CI reported significantly greater increases in expressing verbal appreciation by 0.33 scores (95% CI: 0.04, 0.62) and through action by 0.33 scores (95% CI: 0.03, 0.62) with small effect sizes (Cohen’s d: 0.38–0.39, all *p* < 0.05) at 1-month follow-up, but not at 3-month follow-up.

At the focus-group interviews, participants reported increased family communication because of new common topics (health and exercise) to discuss.

*“I don’t know if this is considered an improvement in communication, but I think it is good. For example, if we don’t do it (ZTEx) very well, then we can say*… *‘hey hey hey hey*… *don’t hold on to it (for support)’*… *I think this is*… *also communication.” (Housewife, female, 45–49 years)*

*“Perhaps our family will have an additional topic to talk about. Maybe normally we wouldn’t discuss exercise with family members*…*but after this exercise and activity, we will have more to talk about with our family members.” (Full-time student, male, 20–24 years)*

### Changes in Family Well-Being

Table 4 shows CI reported significant improvements in family well-being at 1- and 3-month follow-up with small effect size (Cohen’s d: 0.20–0.30; all *p* < 0.05), but no significant changes in family well-being were reported in BI and CAU at 1-month and 3-month follow-up.

**FIGURE 5:**
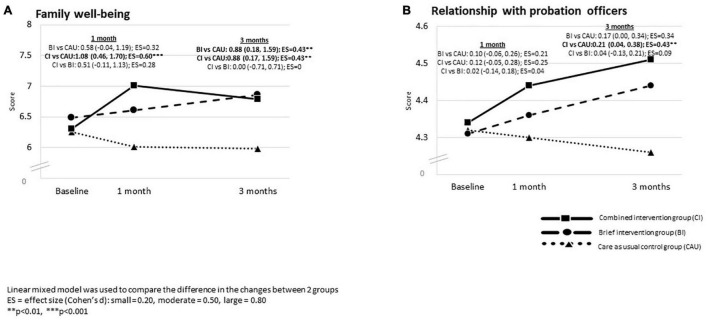
The between–group difference in the changes in family well-being and relationship with probation officers at 1- and 3-month follow-up in three groups: Intention-to-treat analysis.

Figure 5 shows, compared with CAU, BI reported significantly greater improvement in family well-being by 0.88 scores (95% CI: 0.18, 1.59; Cohen’s d: 0.43, *p* < 0.01) at 3-month follow-up, but not at 1-month follow-up. CI reported significantly greater improvements in family well-being by 1.08 scores (95% CI: 0.46, 1.70) and 0.88 scores (95% CI: 0.17, 1.59) than the CAU, with small to moderate effect sizes (Cohen’s d: 0.43–0.60, all *p* < 0.01) at 1- and 3-month follow-up, respectively. No significant difference in changes between BI, and CI were reported at 1- and 3-month follow-up.

At the focus-group interviews, probationers reported improved family relationships and felt happier with their families than before.

*“Ever since I joined this “ZTEx” (activity), my relationship with my son has improved a lot because he is curious when I exercised, and he joined in.”(Housewife, female, 40–44 years)*

*“It (family relationship) has become better. (We) talk more.” (Housewife, female, 40–44 years).*

*“For example, when we do the single-leg stance, if we do it well, then we all praise each other*…*with a lot of smiles. I felt happier and our communication has improved.” (Housewife, female, 45–49 years).*

### Changes in the Relationship Between Probationers and Probation Officers

Table 4 also shows BI and CI reported significant improvements in the relationship between probationers and probation officers with a small effect size at 3-month follow-up (Cohen’s d: 0.21–0.31; all *p* < 0.05), but not at 1-month follow-up. CAU reported no such significant changes at 1- and 3-month follow-up.

Figure 5 shows that the CI reported significantly greater improvement in the relationship between probationers and probation officers than the CAU by 0.21 scores (95% CI: 0.04, 0.38; Cohen’s d: 0.43, *p* < 0.01) with a small effect size at 3-month follow-up, but not at 1-month follow-up. There was no significant difference in the changes in the relationship between probationers and probation officers between BI and CAU and between BI and CI, at 1- and 3-month follow-up.

At the focus-group interviews, participants reported changes in their impressions of probation services, and enhanced trust with probation officers and receiving valuable advice from probation officers.

*“I think it (probation service) was different from what I expected*… *there was some pressure before. But later, I realized that the probation officer is very kind and gave us practical help and advice on our real-life problems.” (Full-time employee, male, 30–34 years)*

*“It is much better to have another person that I can share my thoughts and wants*…. *because I do not want to make my family worry.” (Full-time employee, female, 45–49 years)*

The complete case analyses show similar findings to those of the main analyses (Supplementary Tables 1–4).

### Feedback of the Program Design and Content and Suggestion

At the focus group interviews, probationers provided very positive feedback on using the topic of physical activity to start communication with family members. The workbook and handgrip souvenirs acted as good reminders to do regular exercise and promote positive family communication.

*“I think urban dwellers are very busy*…*this is a way to let them know there are exercises that do not require extra time or a specific location*… and *through this way you know it (ZTEx) improves your health, or you can do it (ZTEx) with your family. Maybe you don’t have time to communicate with your family but you can do exercises together, you don’t need to talk*… *doing it together as a family will be beneficial to family health.” (Full-time employee, female, 20–24 years)*

*“The information is excellent. It (workbook) reminds you when you see it. I can do this, so it’s okay.” (Housewife, female, 65 years or above)*

*“I think it’s really clear*… *with introductions on how to do it (ZTEx). It (workbook) has pictures*… *like how to sit.” (Housewife, female, 45–49 years)*

*“For those who don’t regularly exercise*…*it (souvenir) acts as a motivation.” (Full-time employee, male, 40–44 years)*

The group activity provided an opportunity for valuable family time to do fun activities with family members. The activities served as an ice-breaker to express appreciation to family members under a positive atmosphere and encouragement from probation officers.

*“My favorite section was the exercises (ZTEx). It allows us to learn different types of exercise. Maybe normally you only move your legs a bit, but you don’t know about seated cycling. You probably didn’t know about them (ZTEx) before he talked about them.” (Full-time student, female, 20–24 years)*

*“I think without the group activity acting as a foundation, I may not be as interested in trying it (ZTEx). So, it made both of us enthusiastic during the exercise (ZTEx), and the group activity provided an opportunity to express appreciation to my wife, which I have not done for few years.” (Full-time employee, male, 30–34 years)*

## Discussion

This is the first RCT targeted at probationers. Our holistic health intervention, with simple, lifestyle-integrated physical activity (ZTEx) and the integration of positive psychology themes of “Praise and Gratitude,” not only enhanced probationers’ holistic (physical and psychological) health and family communication and well-being but also their relationships with probation officers.

We have first shown probationers receiving existing probation service (CAU) had enhanced physical activity, fitness performance, psychological health, and family communication with small effect sizes. The probationers who additionally received a brief positive family holistic health intervention integrating physical activity and positive psychology (BI), and those who received both BI and group activity (CI), showed improvements in physical activity, family communication and family well-being. Using CAU as controls, we have shown evidence of the effectiveness of CI in improvements in the relationships with probation officer with a small effect size. CI also showed greater increases in physical activity and family communication than the BI with small to moderate effect sizes. Qualitative feedbacks corroborated the quantitative findings.

Our intervention utilizing simple strength and stamina-enhancing physical activity (ZTEx) is advantageous over other physical activity interventions. It is easy for anyone to start and sustain, requiring no money, equipment or a specified location and can be done anywhere and integrated into everyday life (Lai et al., 2020). By using a foot-in-the-door approach and encouraging probationers to start behavior change in small steps by highlighting the simplicity and benefits of ZTEx, the intervention showed further increases in ZTEx and moderate physical activity among probationers that was similar to the findings in our community-based studies (Lai et al., 2018, 2020; Lai A. et al., 2019; Lai Y. et al., 2019). Additionally, there are some studies on improving health and well-being and reducing psychological distress through exercise among prisoners and those who have committed more serious crimes (Kerekes et al., 2017; Sfendla et al., 2018; Wangmo et al., 2018).

Our findings suggest that integration of physical activity and positive psychology in probation services can enhance personal and family well-being. For probationers’ psychological well-being, there were significant within-group improvements in the three groups (i.e., improved mental quality of life in the CAU and the CI, enhanced self-esteem and subjective happiness in the BI, and reduced anxiety and depression symptoms in the CI). However, we found no significant between-group difference in psychological well-being. This might be due to the intervention being primarily targeted at improving physical activity and family communication, thus more difficult to see the distant effects on psychological well-being.

The utilization of an experiential learning approach, “learning by doing,” and interactive strategies in a group activity is recognized as a powerful teaching and learning tool (Newman et al., 2017), and has been used to explain the learning process of individuals and groups. Practicing exercises together and doing interactive games with probationers and their family members may be particularly beneficial for engaging individuals and providing an essential opportunity to express appreciation and gratitude to family members. This strategy should be better than didactic programs in managing the challenges of rapid engagement and an important component in many behavior change models through practice (Kolb, 2015).

Family communication is crucial for maintaining and promoting strong family relationships (Galvin et al., 2015). Our intervention showed significantly greater improvements in family communication between probationers and their family members in CI than BI and CAU. This may be explained by the group activity experience involving family members of probationers in CI, where they observed and practiced the behaviors of others (role models) on how to express appreciation and concerns to their family members. Seeking comfort and support from family members through advice, encouragement, and affection, which is an effective coping mechanism in combating stressful and negative life events (Chou and Chi, 2001; Thomas et al., 2017). Besides, this intervention enhanced working relationships between probation officers and probationers. Better relationships predict better probation outcomes (Morash et al., 2015; Sloas et al., 2020), and higher perceived helpfulness of probation (De Lude et al., 2012). This improvement may offer a more productive and effective probation service and offer a bigger chance for the probationers to successfully re-integrate into daily life.

Our study had several limitations. First, because validated questionnaires were unavailable, we self-developed our outcome-based questions to assess the probationers’ practices in relation to doing simple strength- and stamina-enhancing physical activity by themselves and with their family members and expressing appreciation to family members. The acceptability and applicability of these questions were shown in our previous studies with similar designs (Ho et al., 2020; Lai et al., 2020). Second, we were unable to assess the accumulated duration of physical activity objectively; we only measured the self-reported number of days engaged in physical activity. Self-reported moderate and vigorous physical activity values can be higher than objectively measured values, particularly in inactive participants (LeBlanc and Janssen, 2010). Third, as the intervention was a community-based intervention and the questionnaires had to be kept short, we could not assess changes in all the cognitive factors for the formation of exercise motivation and regulatory factors for regular physical activity. To further understand how intervention effects can be sustained and maintained for longer periods, future studies should identify specific effective intervention components, and assess changes in cognitive and regulatory factors such as risk perception and self-monitoring. More targeted interventions with specific components on enhancing psychological well-being and with greater involvement of family members could be conducted. Finally, we could not rule out social desirability bias. But as our assessments were anonymous and some outcomes showed no changes, such bias should not be substantial.

To conclude, our trial provided the first evidence of the effectiveness of a brief and preventive positive family holistic health intervention with ZTEx and positive psychology. This low-cost, theory- and community-based intervention, with quantitative and qualitative evaluations, offers a new model incorporating physical activity and positive psychology themes of ‘Praise and Gratitude’ for enhancing probation service to improve probationers’ personal and family well-being and the relationship with probation officers.

## Data Availability Statement

The dataset presented in this article is not readily available because the sharing of data to third parties was not mentioned in subjects’ consent. Requests to access the dataset should be directed to the corresponding author.

## Ethics Statement

The studies involving human participants were reviewed and approved by The Institutional Review Board of The University of Hong Kong/Hospital Authority Hong Kong West Cluster (reference number: UW125-249). Written informed consent to participate in this study was provided by the participants’ legal guardian/next of kin.

## Author Contributions

AL led the conception and design of the study, and carried out the study. AL and SS were responsible for interpreting the data and drafting the manuscript. AL, GC, and T-HL were involved in statistical analysis. AL, SS, CT, AW, SC, and T-HL were closely involved in data interpretation and manuscript revision. All authors read and approved the final manuscript.

## Conflict of Interest

The authors declare that the research was conducted in the absence of any commercial or financial relationships that could be construed as a potential conflict of interest.

## Publisher’s Note

All claims expressed in this article are solely those of the authors and do not necessarily represent those of their affiliated organizations, or those of the publisher, the editors and the reviewers. Any product that may be evaluated in this article, or claim that may be made by its manufacturer, is not guaranteed or endorsed by the publisher.

## Footnotes

1
https://www.youtube.com/user/familyhk3h/videos
