# Supplementary material for: A Randomized Controlled Trial of a Positive Family Holistic Health Intervention for Probationers in Hong Kong: A Mixed-Method Study
Source: Front Psychol. 2021 Dec 7;12:739418. doi: 10.3389/fpsyg.2021.739418 (PMC8689060; doi:10.3389/fpsyg.2021.739418)
Supplement: Supplementary file 1 [file Data_Sheet_1.PDF]

Supplementary Table 1 Baseline demographic characteristics of probationers (n=297)

	All	CAU	BI	CI	
	n =297	n = 99	n = 102	n = 96	p-value
	N (%)	n (%)	n (%)	n (%)	
**Sex**					
Male	143 (51.1)	52 (55.3)	46 (47.9)	45 (50.0)	0.58
Female	137 (48.9)	42 (44.7)	50 (52.1)	45 (50.0)	
**Age**					
12-19	44 (15.1)	21 (21.2)	12 (12.1)	11 (11.7)	0.45
20-39	136 (46.6)	43 (43.4)	47 (47.5)	46 (48.9)	
40-59	81 (27.7)	23 (23.2)	31 (31.3)	27 (28.7)	
≥60	31 (10.6)	12 (12.1)	9 (9.1)	10 (10.6)	
**Marital status** [b]					
Not married	154 (52.9)	57 (57.6)	46 (46.5)	51 (54.8)	0.37
Married	101 (34.7)	33 (33.3)	40 (40.4)	28 (30.1)	
Separated, divorced, widowed	36 (12.1)	9 (9.1)	13 (13.1)	14 (15.1)	
**Education** [c]					
Primary or below	40 (13.7)	12 (12.2)	13 (13.1)	15 (16.0)	0.78
Secondary	195 (67.0)	70 (71.4)	66 (66.7)	59 (62.8)	
Post-secondary or above	56 (19.2)	16 (16.3)	20 (20.2)	20 (21.3)	
**Employment** [d]					
Student	24 (8.4)	14 (14.4)	5 (5.2)	5 (5.4)	0.12
Employed full-time/part-time	186 (65.0)	63 (64.9)	63 (65.6)	60 (64.5)	
Unemployed/retired	27 (9.4)	9 (9.3)	8 (8.3)	10 (10.8)	
Homemaker	41 (14.3)	9 (9.3)	15 (15.6)	17 (18.3)	
**Duration of probation**					
Half year or below	191 (66.6)	63 (64.9)	71 (72.4)	57 (62.0)	0.15
Half year to 1 year	74 (25.8)	22 (22.7)	24 (24.5)	28 (30.4)	
1 year to 1.5 years	20 (7.0)	10 (10.3)	3 (3.1)	7 (7.6)	
>1.5 years	2 (0.7)	2 (2.1)	0 (0.0)	0 (0.0)	

CAU= Care-as-usual group, BI = Brief intervention group, CI = Combined intervention group

[a]p-value for the differences among 3 groups at baseline

[b] 6 missing value, n=291; [c] 6 missing value, n=291; [d] 19 missing value, n=278

Supplementary Table 2 The within-group difference in physical activity, fitness performances, psychological well-being and quality of life at 1- and 3-month follow-up in 3 groups: Complete-case analysis

	CAU (n=99)		BI (n=102)		CI (n=96)	
	**Mean ± SD**	**Cohen's d**	**Mean ± SD**	**Cohen's d**	**Mean ± SD**	**Cohen's d**
**Physical activity**						
**Days spent engaging in physical activity while seated**						
T1	1.9 ± 2.4$^{\#}$		2.5 ± 2.6$^{\#}$		2.2 ± 2.6$^{\#}$	
T2, (T2 vs T1)	2.6 ± 2.7	0.26**	2.9 ± 2.4	0.16	2.9 ± 2.4	0.29*
T3, (T3 vs T1)	2.4 ± 2.4	0.19*	3.3 ± 2.5	0.32**	2.8 ± 2.5	0.26*
**Days spent engaging in physical activity while standing**						
T1	2.2 ± 2.5		2.5 ± 2.6$^{\#}$		2.3 ± 2.6$^{\#\#\#}$	
T2, (T2 vs T1)	2.3 ± 2.5	0.04	3.0 ± 2.5	0.19	3.2 ± 2.5	0.37***
T3, (T3 vs T1)	2.4 ± 2.4	0.10	3.3 ± 2.5	0.32*	3.1 ± 2.5	0.33**
**Days spent engaging in moderate physical activity**						
T1	2.3 ± 2.5		2.5 ± 2.6		1.9 ± 2.2$^{\#\#\#}$	
T2, (T2 vs T1)	2.1 ± 2.4	-0.08	2.3 ± 2.4	-0.09	3.1 ± 2.4	0.52***
T3, (T3 vs T1)	2.4 ± 2.5	0.04	2.2 ± 2.2	-0.15	2.8 ± 2.3	0.38***
**Days spent engaging in vigorous physical activity**						
T1	1.3 ± 1.9		1.4 ± 1.9		1.1 ± 1.6$^{\#}$	
T2, (T2 vs T1)	1.2 ± 1.8	-0.06	1.6 ± 2.0	0.11	1.6 ± 1.9	0.33**
T3, (T3 vs T1)	1.1 ± 1.5	-0.11	1.6 ± 2.0	0.10	1.5 ± 1.8	0.23*
**Physical fitness**						
**Single-leg stand test, seconds**						
T1	86.0 ± 39.9		91.4 ± 40.3		84.2 ± 39.8	
T2, (T2 vs T1)	85.4 ± 38.0	-0.02	90.4 ± 38.0	-0.02	84.1 ± 38.9	0.00
T3, (T3 vs T1)	84.5 ± 38.3	-0.04	90.7 ± 38.2	-0.04	82.7 ± 39.9	-0.01
**30-s chair stand test, number of stands**						
T1	19.0 ± 7.7$^{\#\#\#}$		18.6 ± 7.7$^{\#\#\#}$		22.0 ± 9.0$^{\#}$	
T2, (T2 vs T1)	21.0 ± 9.1	**0.24*** **	21.3 ± 8.2	**0.33*** **	23.3 ± 9.8	**0.13***
T3, (T3 vs T1)	21.9 ± 9.2	**0.34*** **	21.8 ± 8.5	**0.39*** **	23.0 ± 8.7	**0.12***
**Psychological well-being**						
**Self-esteem**						
T1	27.0 ± 4.6		27.0 ± 3.6$^{\#}$		27.8 ± 4.1	
T2, (T2 vs T1)	27.3 ± 4.6	0.08	27.6 ± 3.5	**0.16****	27.8 ± 4.6	0.00
T3, (T3 vs T1)	27.1 ± 4.9	0.02	27.6 ± 3.7	0.14	27.9 ± 4.2	0.01
**Subjective happiness**						
T1	17.1 ± 4.8		17.6 ± 4.1$^{\#\#\#}$		17.7 ± 4.8	
T2, (T2 vs T1)	17.6 ± 4.7	0.11	17.9 ± 4.0	0.07	18.1 ± 4.2	0.10
T3, (T3 vs T1)	17.7 ± 4.2	0.15	18.8 ± 4.1	**0.31*** **	18.1 ± 4.3	0.10
**Anxiety and depression**						
T1	2.4 ± 2.6		3.0 ± 2.9		3.0 ± 3.1$^{\#}$	
T2, (T2 vs T1)	2.6 ± 3.0	0.08	2.7 ± 3.0	-0.07	2.2 ± 2.3	**-0.29****
T3, (T3 vs T1)	2.4 ± 2.6	0.03	2.7 ± 2.9	-0.10	2.4 ± 2.6	**-0.21***
**Life satisfaction**						
T1	20.9 ± 7.3		21.9 ± 7.1		21.8 ± 6.9	
T2, (T2 vs T1)	21.7 ± 7.4	0.10	21.9 ± 6.5	0.00	23.1 ± 6.7	**0.19***
T3, (T3 vs T1)	21.8 ± 7.3	0.12	22.9 ± 6.1	0.15	23.1 ± 6.8	0.20
**Quality of life**						
**Physical quality of life**						
T1	47.4 ± 8.8		47.6 ± 8.4		46.5 ± 8.8	
T2, (T2 vs T1)	46.9 ± 8.4	-0.05	47.9 ± 8.6	0.04	47.5 ± 8.0	0.12
T3, (T3 vs T1)	48.2 ± 7.9	0.10	47.4 ± 8.1	-0.02	47.0 ± 8.2	0.06
**Mental quality of life**						
T1	44.5 ± 9.8$^{\#}$		44.2 ± 8.4		45.1 ± 9.0	
T2, (T2 vs T1)	46.8 ± 10.1	**0.23****	45.1 ± 9.3	0.11	47.0 ± 9.1	**0.22***
T3, (T3 vs T1)	45.5 ± 10.3	0.10	45.2 ± 8.9	0.12	45.9 ± 8.7	0.09

T1 = baseline, T2 = 1 month, T3 = 3 months

Repeated Measures Analysis of Variance and paired t-test to compare parametric data among three timepoints and between two timepoints, respectively.

T2 vs T1 = values at 1 month versus values at baseline; T3 vs T1 = values at 3-months versus values at baseline

Difference among three timepoints: $^{\#}p < .05$, $^{\#\#}p<0.01$, $^{\#\#\#}p<0.001$; Difference between two timepoints: *p < 0.05, **p < 0.01, ***p < 0.001

Effect size (Cohen's d): small = 0.20, moderate = 0.50, and large = 0.80

Supplementary Table 3 The within-group difference in family well-being and relationship with probation officers at 1- and 3-month follow-up in 3 groups: Complete-case analysis

	CAU (n=99)		BI (n=102)		CI (n=96)	
	Mean ± SD	Cohen's d	Mean ± SD	Cohen's d	Mean ± SD	Cohen's d
**Family communication**						
**Did physical activity with family members, score**						
T1	1.7 ± 0.9$^{\#\#\#}$		1.8 ± 1.0$^{\#\#\#}$		1.8 ± 0.9$^{\#\#\#}$	
T2, (T2 vs T1)	1.9 ± 1.0	**0.19***	2.7 ± 1.0	**0.86*****	2.6 ± 1.0	**0.84*****
T3, (T3 vs T1)	2.1 ± 1.0	**0.40*****	2.8 ± 1.1	**0.89*****	2.6 ± 0.9	**0.79*****
**Praised family members to do physical activity, score**						
T1	2.4 ± 1.0		2.3 ± 1.0$^{\#\#\#}$		2.6 ± 1.1$^{\#\#}$	
T2, (T2 vs T1)	2.5 ± 1.0	0.11	2.8 ± 1.0	**0.50*****	2.9 ± 0.9	**0.36****
T3, (T3 vs T1)	2.6 ± 0.9	**0.20***	2.9 ± 0.9	**0.60*****	3.0 ± 1.0	**0.39****
**Discussing the importance of exercises with family, score**						
T1	2.7 ± 1.2		2.6 ± 1.2$^{\#\#\#}$		2.8 ± 1.1$^{\#}$	
T2, (T2 vs T1)	2.7 ± 1.1	-0.04	2.9 ± 1.0	**0.26****	3.0 ± 1.0	0.21
T3, (T3 vs T1)	2.7 ± 1.1	-0.05	3.1 ± 0.9	**0.47*****	3.2 ± 1.0	**0.32****
**Expressed verbal appreciation to family members, score**						
T1	2.9 ± 1.1		3.0 ± 1.1$^{\#}$		3.0 ± 1.0$^{\#\#\#}$	
T2, (T2 vs T1)	2.8 ± 1.1	-0.10	3.0 ± 1.0	0.07	3.3 ± 0.9	**0.33****
T3, (T3 vs T1)	2.9 ± 1.0	0.03	3.2 ± 0.9	**0.27****	3.4 ± 1.0	**0.42*****
**Expressed appreciation through action to family members, score**						
T1	2.9 ± 1.0		3.1 ± 1.1		3.1 ± 1.1$^{\#\#}$	
T2, (T2 vs T1)	2.9 ± 1.0	0.01	3.1 ± 0.9	-0.04	3.4 ± 1.0	**0.30***
T3, (T3 vs T1)	2.8 ± 1.1	-0.08	3.3 ± 1.0	0.18	3.4 ± 1.1	**0.35****
**Family well-being, score**						
T1	6.3 ± 2.5		6.5 ± 2.5		6.4 ± 2.4$^{\#\#}$	
T2, (T2 vs T1)	6.1 ± 2.6	-0.10	6.7 ± 2.5	0.06	7.2 ± 2.3	**0.33*****
T3, (T3 vs T1)	6.0 ± 2.8	-0.11	6.9 ± 2.4	0.16	6.9 ± 2.4	**0.22***
**Relationship with probation officers, score**						
T1	4.3 ± 0.7		4.3 ± 0.7$^{\#}$		4.3 ± 0.7$^{\#\#}$	
T2, (T2 vs T1)	4.3 ± 0.7	-0.03	4.4 ± 0.6	0.09	4.5 ± 0.6	0.18
T3, (T3 vs T1)	4.3 ± 0.7	-0.09	4.5 ± 0.6	**0.21***	4.5 ± 0.6	**0.31****

T1 = baseline, T2 = 1 month, T3 = 3 months

T2 vs T1 = values at 1-month versus values at baseline; T3 vs T1 = values at 3-months versus values at baseline

Repeated Measures Analysis of Variance and paired t-test to compare parametric data among three timepoints and between two timepoints, respectively. Difference among three timepoints: $^{\#}p < .05$, $^{\#\#}p<0.01$, $^{\#\#\#}p<0.001$; Difference between two timepoints: *p < 0.05, **p < 0.01, ***p < 0.001

Effect size (Cohen's d): small =0.02, moderate =0.50, large = 0.80.

Supplementary Table 4 The between-group difference in the changes in physical fitness, psychological well-being, and quality of life at 1- and 3-month follow-up in 3 groups: Complete case analysis

	BI vs CAU		CI vs CAU		CI vs BI	
	Mean difference (95%CI)	Cohen's d	Mean difference (95%CI)	Cohen's d	Mean difference (95%CI)	Cohen's d
**Difference in the changes at 1 month**						
**Physical activity**						
Days spent engaging in Zero-time exercise while seated, days	0.07 (-0.74, 0.87)	0.03	0.18 (-0.63, 0.99)	0.08	0.11 (-0.70, 0.92)	0.05
Days spent engaging in Zero-time exercise while standing, days	0.62 (-0.15, 1.39)	0.28	0.88 (0.10, 1.65)	0.40*	0.26 (-0.52, 1.03)	0.12
Days spent engaging in moderate physical activity, days	0.18 (-0.54, 0.90)	0.09	1.42 (0.70, 2.15)	0.70***	1.25 (0.52, 1.98)	0.62***
Days spent engaging in vigorous physical activity, days	0.39 (-0.21, 0.99)	0.23	0.65 (0.05, 1.25)	0.39*	0.26 (-0.35, 0.86)	0.15
**Physical fitness**						
Single-leg stand test, second	1.49 (-7.73, 10.71)	0.06	0.28 (-9.12, 9.69)	0.01	-1.21 (-10.61, 8.19)	-0.05
30-s chair stand test, no. of stand	0.42 (-1.36, 2.20)	0.08	-0.71 (-2.56, 1.15)	-0.14	-1.13 (-2.97, 0.72)	-0.22
**Psychological well-being**						
Self-esteem	0.21 (-0.82, 1.24)	0.07	-0.18 (-1.23, 0.87)	-0.06	-0.39 (-1.44, 0.66)	-0.13
Subjective happiness	0.06 (-1.01, 1.12)	0.02	0.21 (-0.88, 1.30)	0.07	0.15 (-0.93, 1.23)	0.05
Anxiety and depression	-0.23 (-1.07, 0.62)	-0.09	-0.82 (-1.68, 0.04)	-0.35	-0.60 (-1.45, 0.26)	-0.25
Life satisfaction	-0.06 (-1.75, 1.63)	-0.01	0.95 (-0.77, 2.66)	0.20	1.01 (-0.69, 2.71)	0.21
**Quality of life**						
Physical quality of life	0.86 (-1.24, 2.96)	0.14	1.39 (-0.73, 3.52)	0.24	0.54 (-1.61, 2.68)	0.09
Mental quality of life	-1.54 (-4.13, 1.06)	-0.21	-0.26 (-2.90, 2.37)	-0.04	1.27 (-1.38, 3.92)	0.17
**Family communication**						
Did physical activity with family members, score	0.77 (0.45, 1.08)	0.86***	0.71 (0.39, 1.03)	0.79***	-0.05 (-0.37, 0.26)	-0.06
Praised family members to do physical activity, score	0.37 (0.06, 0.68)	0.42*	0.40 (0.08, 0.71)	0.45**	0.03 (-0.29, 0.34)	0.03
Expressed verbal appreciation to family members, score	0.22 (-0.08, 0.52)	0.26	0.52 (0.22, 0.82)	0.61***	0.30 (0.00, 0.60)	0.35
Expressed appreciation to family members through action, score	0.12 (-0.19, 0.42)	0.13	0.42 (0.11, 0.72)	0.48**	0.30 (-0.01, 0.61)	0.35
**Family well-being**	0.61 (-0.02, 1.25)	0.34	1.17 (0.53, 1.81)	0.65***	0.56 (-0.09, 1.20)	0.31
**Relationship with probation officers**	0.09 (-0.08, 0.26)	0.19	0.12 (-0.05, 0.29)	0.25	0.03 (-0.14, 0.20)	0.06
**Difference in the changes at 3 months**						
**Physical activity**						
Days spent engaging in Zero-time exercise while seated, days	0.70 (-0.10, 1.50)	0.31	0.34 (-0.46, 1.15)	0.15	-0.36 (-1.16, 0.44)	-0.16
Days spent engaging in Zero-time exercise while standing, days	0.75 (-0.03, 1.54)	0.34	0.60 (-0.19, 1.40)	0.27	-0.15 (-0.95, 0.64)	-0.07
Days spent engaging in moderate physical activity, days	-0.37 (-1.11, 0.38)	-0.18	0.66 (-0.09, 1.40)	0.31	1.03 (0.27, 1.78)	0.49**
Days spent engaging in vigorous physical activity, days	0.46 (-0.11, 1.03)	0.29	0.53 (-0.04, 1.10)	0.33	0.07 (-0.50, 0.65)	0.05
**Physical fitness**						
Single-leg stand test, second	0.95 (-8.87, 10.76)	0.03	-0.15 (-10.12, 9.83)	-0.01	-1.09 (-11.06, 8.87)	-0.04
30-s chair stand test, number of stand	0.01 (-1.78, 1.79)	0.00	-1.54 (-3.39, 0.32)	-0.30	-1.54 (-3.39, 0.31)	-0.30
**Psychological well-being**						
Self-esteem	0.52 (-0.52, 1.55)	0.18	0.28 (-0.78, 1.33)	0.09	-0.24 (-1.29, 0.81)	-0.08
Subjective happiness	0.77 (-0.33, 1.88)	0.25	0.16 (-0.97, 1.29)	0.05	-0.61 (-1.73, 0.50)	-0.20
Anxiety and depression	-0.10 (-0.92, 0.71)	-0.04	-0.35 (-1.18, 0.48)	-0.15	-0.24 (-1.07, 0.58)	-0.11
Life satisfaction	0.38 (-1.40, 2.16)	0.07	0.95 (-0.86, 2.75)	0.19	0.57 (-1.22, 2.36)	0.11

**Quality of life**						
Physical quality of life	-0.32 (-2.55, 1.91)	-0.05	0.05 (-2.21, 2.31)	0.01	0.37 (-1.91, 2.64)	0.06
Mental quality of life	-0.12 (-2.82, 2.58)	-0.02	-0.19 (-2.93, 2.55)	-0.03	-0.08 (-2.83, 2.68)	-0.01
**Family communication**						
Did physical activity with family members, score	0.64 (0.29, 0.99)	0.64***	0.44 (0.09, 0.80)	0.45**	-0.20 (-0.55, 0.16)	-0.20
Praised family members to do physical activity, score	0.37 (0.05, 0.69)	0.41*	0.40 (0.08, 0.72)	0.45**	0.03 (-0.29, 0.35)	0.04
Expressed verbal appreciation to family members, score	0.25 (-0.06, 0.56)	0.29	0.48 (0.17, 0.80)	0.55**	0.23 (-0.09, 0.54)	0.26
Expressed appreciation to family members through action, score	0.35 (0.02, 0.69)	0.37*	0.57 (0.23, 0.91)	0.60***	0.22 (-0.12, 0.56)	0.23
**Family well-being, score**	0.94 (0.20, 1.68)	0.45**	0.96 (0.21, 1.71)	0.46**	0.02 (-0.73, 0.78)	0.01
**Relationship with probation officers**	0.16 (-0.01, 0.34)	0.32	0.21 (0.03, 0.39)	**0.41***	0.04 (-0.14, 0.22)	0.09

CAU= Care-as-usual group, BI = Brief intervention group, CI = Combined intervention group
Linear mixed model was adopted to examine the between-group difference
Effect size (Cohen's d): small =0.02, moderate =0.50, large = 0.80; * $p < 0.01$
